# *ARCIMBOLDO* on coiled coils

**DOI:** 10.1107/S2059798317017582

**Published:** 2018-03-02

**Authors:** Iracema Caballero, Massimo Sammito, Claudia Millán, Andrey Lebedev, Nicolas Soler, Isabel Usón

**Affiliations:** a Structural Biology Unit, Institute of Molecular Biology of Barcelona (IBMB–CSIC), Baldiri Reixac 15, 08028 Barcelona, Spain; b CCP4, STFC Rutherford Appleton Laboratory, Research Complex at Harwell, Didcot OX11 0FA, England; c ICREA, Pg. Lluís Companys 23, 08010 Barcelona, Spain

**Keywords:** *ARCIMBOLDO*, coiled coils, phasing, *SHELXE*, *Phaser*

## Abstract

*ARCIMBOLDO* solves the phase problem by combining the location of small model fragments using *Phaser* with density modification and autotracing using *SHELXE*. Mainly helical structures constitute favourable cases, which can be solved using polyalanine helical fragments as search models. Nevertheless, the solution of coiled-coil structures is often complicated by their anisotropic diffraction and apparent translational noncrystallographic symmetry. Long, straight helices have internal translational symmetry and their alignment in preferential directions gives rise to systematic overlap of Patterson vectors. This situation has to be differentiated from the translational symmetry relating different monomers. *ARCIMBOLDO_LITE* has been run on single workstations on a test pool of 150 coiled-coil structures with 15–635 amino acids per asymmetric unit and with diffraction data resolutions of between 0.9 and 3.0 Å. The results have been used to identify and address specific issues when solving this class of structures using *ARCIMBOLDO*. Features from *Phaser* v.2.7 onwards are essential to correct anisotropy and produce translation solutions that will pass the packing filters. As the resolution becomes worse than 2.3 Å, the helix direction may be reversed in the placed fragments. Differentiation between true solutions and pseudo-solutions, in which helix fragments were correctly positioned but in a reverse orientation, was found to be problematic at resolutions worse than 2.3 Å. Therefore, after every new fragment-placement round, complete or sparse combinations of helices in alternative directions are generated and evaluated. The final solution is once again probed by helix reversal, refinement and extension. To conclude, density modification and *SHELXE* autotracing incorporating helical constraints is also exploited to extend the resolution limit in the case of coiled coils and to enhance the identification of correct solutions. This study resulted in a specialized mode within *ARCIMBOLDO* for the solution of coiled-coil structures, which overrides the resolution limit and can be invoked from the command line (keyword coiled_coil) or *ARCIMBOLDO_LITE* task interface in *CCP*4*i*.

## Abstract 2

The *ARCIMBOLDO* method of phasing through the location of small fragments combined with density modification and autotracing is particularly suited to helical structures, but coiled coils remain challenging. Features designed for solving coiled coils at resolutions of up to 3 Å were tested on a pool of 150 structures.

## 1. Introduction   

The phase problem is central to crystallography, and in the case of macromolecular crystals it is often not trivial to solve (Hendrickson, 2013 ▸). Starting phases for the structure factors that are missing from the results of a diffraction experiment are initially approximated by experimental phasing through heavy-atom derivatives or anomalous scattering at particular wavelengths (Hendrickson, 1991 ▸) or using previous structural knowledge from a similar structure in the method of molecular replacement (Rossmann, 1972 ▸; Navaza, 1994 ▸; Read, 2001 ▸). In chemical crystallography, molecules with less than 200 atoms that diffract to atomic resolution are routinely solved *ab initio* from the native diffraction intensities alone by direct methods (Karle & Hauptman, 1956 ▸; Woolfson, 1987 ▸). Small proteins of up to 1000 atoms that diffract to atomic resolution can also be phased by direct methods using the *Shake-and-Bake* algorithm (Miller *et al.*, 1993 ▸; Sheldrick *et al.*, 2012 ▸). Restrictions on data quality and structure size can be relaxed by means of various techniques. These include sophisticated use of the Patterson function (Caliandro *et al.*, 2008 ▸), the use of expected values of structure amplitudes outside the actual resolution limit of the experimental data (Caliandro *et al.*, 2005 ▸; Usón *et al.*, 2007 ▸) and high-resolution density-modification algorithms such as low-density elimination (Shiono & Woolfson, 1992 ▸; Refaat & Woolfson, 1993 ▸), the sphere of influence (Sheldrick, 2002 ▸) and *VLD* (Burla *et al.*, 2012 ▸). Small but highly accurate substructures can provide starting phases leading to successful phasing through density modification, as has been shown with *ACORN* (Foadi, 2003 ▸). As little as 10% of the main-chain atoms may suffice to solve a structure at 2 Å resolution (Millán *et al.*, 2015 ▸). Thus, the atomicity constraints that are essential to direct methods can be substituted by enforcing secondary- or tertiary-structure stereochemistry. A related proof of principle was established using α-helices (Glykos & Kokkinidis, 2003 ▸) or nucleotides (Robertson & Scott, 2008 ▸; Robertson *et al.*, 2010 ▸) as fragments to seed phasing. *ARCIMBOLDO* (Rodríguez *et al.*, 2009 ▸, 2012 ▸) solves structures by combining the search for small polyalanine-model fragments with *Phaser* (McCoy *et al.*, 2007 ▸) with expansion to a fairly complete structure through density modification and autotracing with *SHELXE* (Thorn & Sheldrick, 2013 ▸). Depending on the complexity of the case, a single-multicore workstation may suffice or a grid of computers may be needed (Sammito *et al.*, 2015 ▸). Extremely successful approaches based on more complete models of lower accuracy (Rigden *et al.*, 2008 ▸) have been developed based on the improvement of models derived from remote homologues or *de novo* model generation using *ROSETTA* (Qian *et al.*, 2007 ▸) or *QUARK* (Xu & Zhang, 2012 ▸) combined with molecular replacement with *Phaser* (Read & McCoy, 2016 ▸) or *MOLREP* (Vagin & Teplyakov, 1997 ▸; Vagin & Teplyakov, 2010 ▸). This design underlies methods such as *MR-Rosetta* (DiMaio *et al.*, 2011 ▸), *AMPLE* (Bibby *et al.*, 2012 ▸, 2013 ▸; Keegan *et al.*, 2015 ▸) and other implementations (Shrestha *et al.*, 2011 ▸; Shrestha & Zhang, 2015 ▸).

In general, all-helical structures are favourable cases for phasing with *ARCIMBOLDO*, where polyalanine helices constitute ideal search fragments as they are constant, rigid and nearly ubiquitous. In coiled coils, several amphipathic α-helices are brought together and wound around each other, building a supercoil. The sequences underlying this fold contain characteristic repeats of seven residues leading to left-handed coiling or 11 residues in the case of right-handed coiling (Lupas & Gruber, 2005 ▸). Structures of this kind form an important part of structural studies, as they are found in a variety of proteins involved in diverse cellular processes comprising transcription, ATP synthesis, intracellular transport, transmembrane signalling, membrane fusion and re­modelling, proteostasis and the formation of the extracellular matrix and several cytoskeletal and nuclear structures of the eukaryotic cell (Baxevanis & Vinson, 1993 ▸; Kuhn *et al.*, 2014 ▸). Coiled coils also play a key role in the association of proteins into functional quaternary assemblies. It has often been noted that despite their apparent simplicity, their solution by molecular replacement is challenging (Franke *et al.*, 2011 ▸; Blocquel *et al.*, 2014 ▸; Dauter, 2015 ▸).

Phasing of coiled-coil crystal structures with fragments has been implemented in the *AMPLE* (Thomas *et al.*, 2015 ▸) and *CCsolve* (Rämisch *et al.*, 2015 ▸) pipelines, which combine *de novo* structure prediction (Das *et al.*, 2009 ▸), MR search and, finally, autotracing (Sheldrick, 2010 ▸) or automated model building (Terwilliger *et al.*, 2008 ▸).

In the present work, we have explored fragment phasing on a pool of 150 coiled coils and the results have been used to identify hurdles and equip *ARCIMBOLDO* (Millán *et al.*, 2015 ▸) with a specialized protocol with optimized values of parameters for coiled-coil structures. This coiled_coil mode in *ARCIMBOLDO_LITE* incorporates a new search algorithm to probe and verify alternative helix directions. It relies on advances in the MR search (Oeffner *et al.*, 2018 ▸) and autotracing (Usón & Sheldrick, 2018 ▸). The results of our tests show that the new mode substantially extends the range of data suitable for fragment phasing of coiled-coil structures, and thus the high-resolution limit has been extended from 2.5 Å for the general mode of *ARCIMBOLDO_LITE* to 3.0 Å for the coiled_coil mode. The program is distributed for Linux and MacOS from http://chango.ibmb.csic.es/ARCIMBOLDO_LITE (standalone version) as well as through *CCP*4.

## 2. Materials and methods   

### 2.1. Computing setup   

The tests were run on the eight identical eight-core machines of an HP ProLiant BL460c blade system, using them as single, independent workstations with dual quad-core Xeon E5440 processors at 2.83 GHz with 16 GB RAM and with the Debian GNU/Linux 8.4 operating system. *ARCIMBOLDO_LITE* adjusts the calculations to the available hardware, so that a problem which failed to be solved on a given setup might have been solved on a more powerful workstation or on a grid. Additional tests were run on a machine with two 12-core Xeon processors (E5-2680; 2.5 GHz and 128 GB RAM) and on a grid with HTCondor v.8.4.5 (Tannenbaum *et al.*, 2001 ▸) integrated by a maximum of 160 nodes adding up to 250 GFlops.

### 2.2. Software versions and figures of merit used   

The *ARCIMBOLDO_LITE* binary is deployed for Linux and Macintosh and can be downloaded from our website (http://chango.ibmb.csic.es/). It was generated with PyInstaller 3.2 and Python 2.7.*x*. It requires *Phaser* v.2.7 or higher, which is available from the *PHENIX* (Adams *et al.*, 2010 ▸) distribution, and the latest version of *SHELXE* (Usón & Sheldrick, 2018 ▸) available from the *SHELX* website. Alternatively, *ARCIMBOLDO* and both dependencies can be installed as components of *CCP*4 release 7.0 (Winn *et al.*, 2011 ▸).

Model and maps were examined with *Coot* v.0.8.7 (Emsley *et al.*, 2010 ▸). Figures were prepared with the *PyMOL* molecular-graphics system (v.1.2r2; Schrödinger) and *Matplotlib* v.1.5.3 (Hunter, 2007 ▸). *XPREP* v.2015/1 was used for data analysis (Sheldrick, 2001 ▸). *Phenix.xtriage* from the *PHENIX* distribution was used to calculate the anisotropy eigenvalues.

The figures of merit used in decision making were *Phaser*’s intensity-based log-likelihood gain (LLG; Read & McCoy, 2016 ▸) and the correlation coefficient between observed and calculated normalized intensities (CC; Fujinaga & Read, 1987 ▸) calculated by *SHELXE* (Sheldrick, 2002 ▸). Structure-amplitude-weighted mean phase errors (wMPE; Lunin & Woolfson, 1993 ▸) were calculated with *SHELXE* against the models available from the PDB to assess performance.

### 2.3. Test sets used   

In this study, two pools of coiled-coil crystal structures from the PDB (Bernstein *et al.*, 1977 ▸; Berman *et al.*, 2000 ▸) were used.

The first pool was selected from a previous study (Thomas *et al.*, 2015 ▸) and comprises 94 cases with resolutions ranging between 0.9 and 2.9 Å and sizes between 15 and 618 residues distributed in the asymmetric unit in one to four chains that belong to 32 different space groups in which *C*2 predominates, followed by *P*2_1_2_1_2_1_ and *P*2_1_. They were deposited in the PDB between 1997 and 2012.

One of the structures, PDB entry 3azd, has deposited data with resolution spanning 2.7–0.9 Å but lacks all lower resolution data, and over half of the deposited sigmas are zero. Eight structures, PDB entries 1s9z, 2pnv, 3h00, 3h7z, 3ra3, 3s0r, 3v86 and 4dzk, are merohedrally twinned.

Furthermore, this test set was expanded with a second pool of 56 structures selected from the PDB in the resolution range 2–3 Å with sizes spanning 45–635 amino acids in one to 12 chains. These structures, which were deposited in the years 2001–2016, belong to 26 different space groups, with *P*2_1_, *C*2 and *P*2_1_2_1_2_1_ predominating. Three of them, PDB entries 3miw, 4bl6 and 5ajs, are merohedrally twinned.

The joint set thus covered 0.9–3 Å resolution, asymmetric unit contents ranging from 15 to 635 amino acids and 38 different space groups. No isomorph structures were included, although PDB entries 3mqc and 3nwh are closely related. Table 1 ▸ characterizes both test sets. The PDB codes for all 150 structures are given in Appendix *A* [App appa]. The details of the individual test cases and their PDB entries are presented in the Supporting Information as a table.

## 3. Results and discussion   

### 3.1. Implementation of a graphical user interface   

*ARCIMBOLDO* is distributed in two alternative ways: (i) as a standalone bundle with most dependencies included and (ii) as part of the *CCP*4 program suite starting from release 7.0. Program execution from the command line, which also assumes manual editing of the parameter file, is available in both distributions. The *CCP*4 distribution additionally offers separate task interfaces in *CCP*4*i* for *ARCIMBOLDO_LITE*, *ARCIMBOLDO_BORGES* and *ARCIMBOLDO_SHREDDER* (Sammito *et al.*, 2013 ▸, 2014 ▸, 2015 ▸). It displays key parameters as widgets and also allows the manual input of advanced parameters.

The three *ARCIMBOLDO* programs generate reports in HTML format, which include a list of all of the program parameters (including default parameters), tables characterizing partial or final solutions, and links to the model and map files corresponding to the current best solution. The tables are dynamically updated and are sortable by column values. The corresponding *CCP*4*i* task interfaces have their own simple report pages, through which a user can open the HTML reports in the system default web browser and the best model and corresponding maps in *Coot* (Emsley *et al.*, 2010 ▸).

The parameters that were found to be determinant for solving coiled-coil structures and their optimal values can now be all invoked by a single *ARCIMBOLDO* keyword named coiled_coil, or *via* a checkbox in the *CCP*4*i* *ARCIMBOLDO_LITE* task interface.

### 3.2. Timing benchmarks on various hardware   

*ARCIMBOLDO_LITE* jobs in coiled_coil mode typically took a few hours (5 min for PDB entry 1s9z searching for one helix of 18 amino acids to 19 h for PDB entry 5jxc searching for 12 helices of 18 residues) for cases at resolutions of better than 2 Å on the eight-core machines described above. Lower resolution cases required more intensive computations owing to helix-orientation reversion and verification of potential solutions, which proved to be critical for ruling out false positives.

### 3.3. Overall performance   

**Figure 1:**
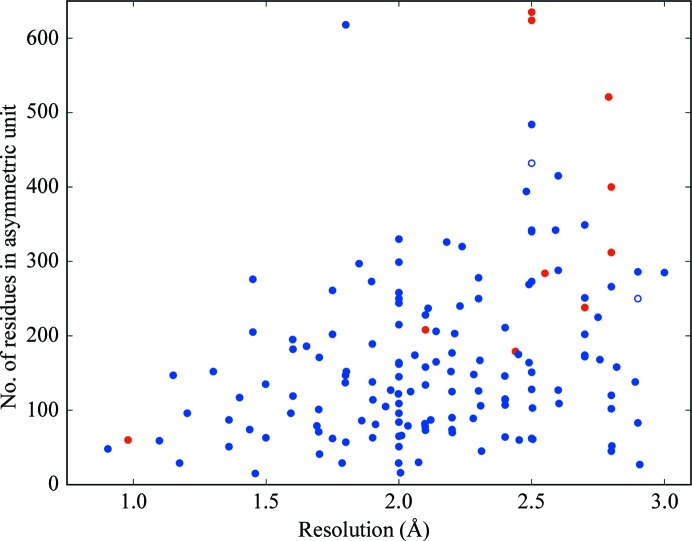
Performance of *ARCIMBOLDO_LITE* on a pool of 150 coiled-coil test structures. A total of 140 structures, corresponding to 93% of the cases, were solved. 137 structures (91%) were phased on the eight-core machines with *ARCIMBOLDO_LITE* and are represented by blue dots. Open dots mark cases where more powerful hardware (a 24-core workstation) was required. The red dots mark the ten unsolved cases. Abscissa represent the resolution and ordinates represent the asymmetric unit content characterizing the test cases.

Fig. 1 ▸ summarizes the single-workstation performance of *ARCIMBOLDO_LITE* on a set of test structures. 94 structures in this test came from a previous study (Thomas *et al.*, 2015 ▸). The lower resolution range was supplemented with a further 28 structures at 2.0–2.5 Å resolution and 28 at 2.5–3.0 Å resolution. For the purpose of this study, a structure was considered to be solved when the weighted mean phase error *versus* the reference deposited with the PDB was below 60°. The percentage of unsolved structures for the first pool was 4.25% (four in 94) and that for the second pool was 10.7% (six in 56). The unsolved structures do not share a common characteristic, but they include cases with issues beyond a typical coiled coil. PDB entry 3azd shows an alarming validation report, with very high clashscore and poor side-chain geometry. Furthermore, its deposited data are extremely incomplete and half of the data have the associated sigmas set to zero. PDB entry 4pna could not be solved, but PDB entry 5f2y (not in our test set), a point mutant of the same protein in space group *I*2 that diffracted to the same resolution, was solved straight away. PDB entry 3s4r also has completeness issues and severe anisotropy. PDB entries 3iv1, 1u4q, 4xa3, 2fxm, 3tul, 2jee and 3mqc all diffracted to 2.5 Å resolution or worse. From these, the larger structures PDB entries 1u4q, 3iv1 and 3tul, with more than 400 residues in the asymmetric unit, are characterized by an expected LLG (McCoy *et al.*, 2017 ▸) of 11 or less for the placement of a helix of 30 residues; thus, it is not surprising that they cannot be solved on a workstation even with ideal data calculated from the model to the experimental resolution. The unsolved PDB entry 3mqc, at 2.8 Å resolution, is close to isostructural to the solved PDB entry 3nwh, although the former contains a somewhat longer construct. A *Microsoft Excel* table deposited as Supporting Information condenses the characteristics and results for each of the structures probed. In total, of the 150 structures, 140 (93%) were solved.

An initial baseline to identify easy-to-solve cases was set by running *ARCIMBOLDO_LITE* with general default parameters on the pool of 150 structures, with the fragment search configured to find four polyalanine helices of 18 residues and using the standard resolution-dependent *SHELXE* parameterization (Sammito *et al.*, 2015 ▸). This straightforward approach was successful in 78 of the 150 cases and led to the identification of the most interesting cases. In general, the choice of search fragments is based on the secondary-structure prediction for the contents of the asymmetric unit and the signal that can be expected from a fragment of given size for the particular data (McCoy *et al.*, 2017 ▸). Furthermore, some trial and error may be necessary, as seen in a case where the effect of helix length was systematically tested (Schoch *et al.*, 2015 ▸).

The following sections describe the particular problems that prevented some of the remaining 72 structures from being immediately solved, and solutions for these problems, which led to phasing solutions in a further, previously unsuccessful, 62 cases.

#### 3.3.1. R.m.s.d. and VRMS   

The 14-residue polyalanine helix typically used in *ARCIMBOLDO_LITE* generally fits helices in target structures with a low r.m.s.d., and a default value of 0.2 Å is set for the fragment search in *Phaser*. Longer helices were used in most of the test cases and the accumulated curvature in coiled coils was expected to lead to higher deviations, but in practice all structures but one were solved by setting the r.m.s.d. to 0.2 Å. PDB entry 3thf in space group *P*2_1_2_1_2 with 349 independent residues at 2.7 Å resolution was only solved by increasing the r.m.s.d. to 0.5 Å.

In *Phaser*’s rigid-group refinement step the input r.m.s.d. parameter can be refined in order to maximize the LLG (Oeffner *et al.*, 2013 ▸) through the variance root-mean-square calculation (VRMS). In solved structures, the VRMS refined to values around 0.1 Å, ranging from 0.05 to 0.53 Å. This roughly corresponds to the default r.m.s.d. parameterization, and therefore refining the r.m.s.d. as a parameter does not have a large effect. The only exception was noted for PDB entry 3v86 at 2.91 Å resolution, where the correct substructure was only discriminated by refining the r.m.s.d. All other cases were insensitive to switching this parameter on or off. As it has not been observed to have negative effects in any case, this calculation is activated by default in the coiled_coil mode.

#### 3.3.2. Translational noncrystallographic symmetry   

**Figure 2:**
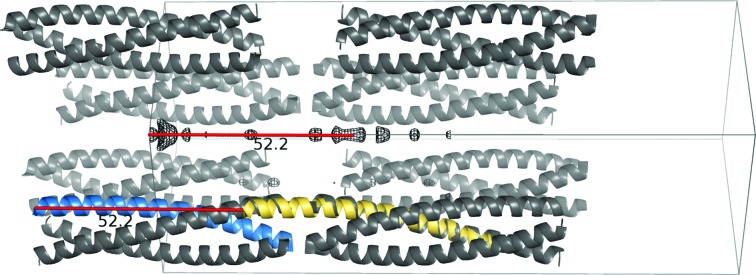
Apparent translational noncrystallographic symmetry in the case of PDB entry 3p7k. The structure is shown as a blue cartoon, with symmetry equivalents as a grey cartoon and the Patterson map contoured at 2σ as a black mesh. The yellow helix corresponds to PDB entry 3p7k translated 52.2 Å by the vector corresponding to the Patterson function peak. It coincides with different portions of symmetry-related helices.

The presence of translational noncrystallographic symmetry (tNCS) is deduced by *Phaser* from the presence of peaks separated from the origin by more than 15 Å and above 20% of the origin peak in the Patterson function calculated using data from 10 to 5 Å resolution. If tNCS is identified, *Phaser* will correct the effect of the modulation in the input data and search for pairs of molecules (groups in a more general case) related by the tNCS vector (Sliwiak *et al.*, 2014 ▸). Parameters describing the translation and small rotation differences between copies are determined and used to compute correction factors to the target function (Read *et al.*, 2013 ▸). By default, *ARCIMBOLDO* makes use of this feature in *Phaser*, simultaneously placing tNCS-related copies associated with a given rotation. This behaviour can be disabled through the instruction .bor file or the *CCP*4*i* interface. The coiled_coil keyword entails its deactivation. As illustrated in Fig. 2 ▸, the internal periodicity of a single helix along with the accidental overlap of vectors derived from the systematic alignment of helices along predominant directions gives rise to strong peaks in the Patterson function (Urzhumtsev *et al.*, 2016 ▸). Thus, PDB entry 3p7k in space group *P*6_3_22 at 2.3 Å resolution, the packing of which is shown in Fig. 2 ▸, contains a single, curved helix of 45 amino acids in the asymmetric unit. Displacing it 52.2 Å in the direction of the *c* axis partially superimposes it on two symmetry equivalents, one of them in the reversed direction. The corresponding Patterson peak displayed in the figure is the maximum identified by *Phaser*, but generating pairs of helices related by such a translation would in this case prevent the finding of a correct solution. Thus, to solve this structure the pairwise placement feature needs to be turned off.

Within the first pool of 94 structures, 19 cases show peaks in the Patterson function which would trigger tNCS pairwise location. Of these, PDB entries 1byz, 1g1j, 1kyc, 1nkd, 1p9i, 1x8y, 1yod, 2b22, 2bez, 2ic6, 2wpq, 3bas, 3hfe, 3k9a, 3m91, 3p7k, 3v86 and 3vgy have been solved, while 3mqc remains unsolved.

Within the second, lower resolution pool of 56 structures, tNCS was identified from the Patterson function in ten solved cases: PDB entries 2ahp, 3efg, 3r3k, 5c9n, 1unx, 2wz7, 1w5h, 2o1j, 3v2r and 3nwh. A further three cases, PDB entries 3iv1, 3tul and 4pna, remain unsolved.

All of these structures were tried with pairwise placement turned off (keyword tNCS:False); that is, placing single helices sequentially as well as placing pairs of tNCS-related helices. In 17 cases a solution was only found by placing single-fragment copies sequentially, whereas pairs of fragments placed as related by the translation vector derived from the Patterson map were either misplaced despite their high scores or discarded at the packing check because of partial overlap with symmetry equivalents. In eight cases, either setting led to a correct solution. In the cases of PDB entries 1g1j, 2o1j and 3nwh, which present true intramolecular tNCS, phasing was only successful by placing tNCS-related pairs. As differentiating genuine intermolecular tNCS from Patterson artefacts is difficult, the default behaviour in *ARCIMBOLDO* for coiled coils will be to avoid the tNCS-related search, but if no solution is achieved this alternative should be tried.

#### 3.3.3. Packing filter at translation search   

Partially overlapping solutions are usually discarded after the translation search. In space groups where proper rotational symmetry operations are present, a recurrent problem is that helices placed on pure rotation axes may be characterized by extremely high LLG scores, while correct solutions may be well below 75% of these values. In all space groups, a second helix placed on top of a previous helix may also lead to disproportionately high scores. In this case, no solution with feasible packing will be output in the list of translation-function solutions, and the process halts as the packing filter discards everything. This recurring problem in helical fragment searches can be overcome by using *Phaser*’s new packing filter within the analysis of the translation function (Oeffner *et al.*, 2018 ▸). This ensures that the top solution used as the reference for selection will not be rejected later in the packing check. *ARCIMBOLDO* uses a very stringent default for either check, allowing no overlap at all.

The cases of PDB entries 2v71 in space group *C*2, 1d7m in *C*222_1_, 4bl6 in *P*6_1_, 3miw in *P*4_2_, 5jxc in *P*2_1_, 3r47 in *P*4_2_, 4bry in *I*4_1_22 and 3thf in *P*2_1_2_1_2 could only be solved when *ARCIMBOLDO* was run activating *Phaser*’s packing filter at translation. The only drawback is an increase in running time, but for coiled coils activating this option is the default, as this issue frequently hinders solution, especially at resolutions worse than 2 Å.

### 3.4. Performance of *ARCIMBOLDO* at resolutions between 2.0 and 3.0 Å   

From the outset, it became evident that lower resolution posed particular difficulties. This prompted us to extend the original test set with 56 structures at worse than 2.0 Å resolution to give a total of 106. Among them, 43 corresponded to resolutions between 2.5 and 3.0 Å (15 structures in the first set and 28 in the second). Eight of the ten structures that remain unsolved correspond to the lower resolution span. PDB entry 3s4r has data to a limit of 2.44 Å resolution but the data are only 85% complete, while the remaining seven data sets are at 2.5 Å resolution or worse.

#### 3.4.1. Reversed helices   

**Figure 3:**
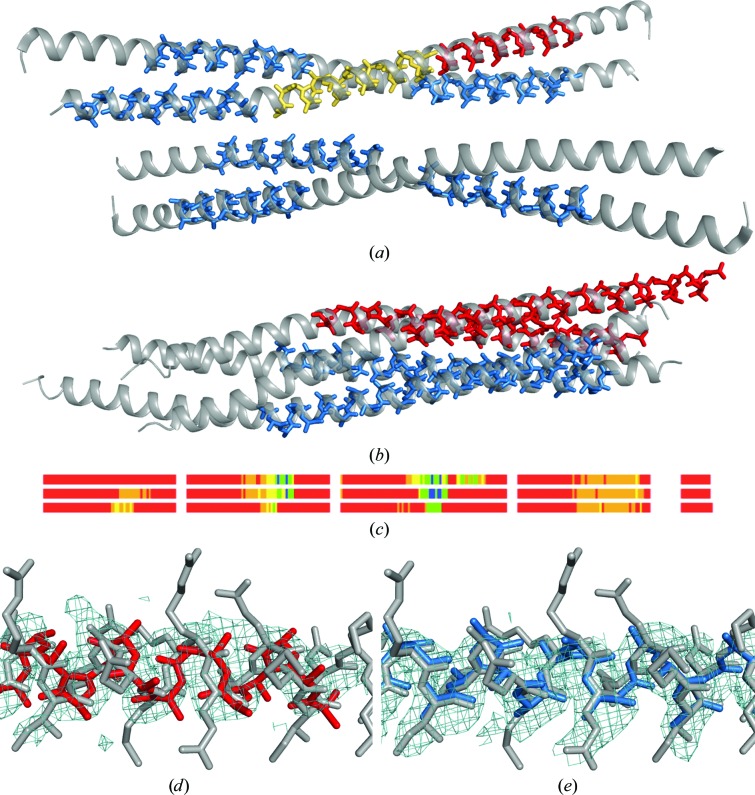
Helices placed reversed. Blue helices are correctly placed; red and yellow helices are reversed. (*a*) PDB entry 2jee at 2.8 Å resolution; fragments placed, shown as sticks, are superimposed on the origin-shifted PDB structure shown as a grey cartoon. (*b*) PDB entry 2nps at 2.5 Å resolution. (*c*) Lack of progress in three cycles of *SHELXE* autotracing of PDB entry 2nps. The first four blocks represent the length of the polypeptide chains, with the r.m.s.d. of the traces colour-coded from blue (<0.3 Å r.m.s.d.), green (<0.6 Å r.m.s.d.) and yellow (<1.0 Å r.m.s.d.) to red, where no trace can be matched within 2.0 Å r.m.s.d. The fifth block represents the length of traced residues that cannot be assigned to any part of the correct structure. The consistent orange-coloured sections, indicating up to 2.0 Å r.m.s.d., correspond to persistent reversed traces. (*d*) Electron-density map contoured at 1σ after density modification and autotracing of an inverted helix in the solution for PDB entry 3miw with errors. (*e*) Electron density for the same region in the correct structure.

At resolutions worse than 2.3 Å it was frequently observed that placement of the first helices occasionally took place in the correct position but in a reversed direction. In the cases of PDB entry 2jee at 2.8 Å resolution and 3miw this issue prevented solution of the structure using the eight-core workstations, whereas in the cases of PDB entries 2nps at 2.5 Å resolution, 3p7k at 2.3 Å resolution and 3h7z at 2.5 Å resolution co­existing correct substructures led to a full solution, even though some of the substructures with reversed helices were sent to expansion as well. The cases of PDB entries 2nps at 2.5 Å resolution with two out of four reversed helices and 2jee at 2.8 Å resolution displaying six correctly located and two reversed helical fragments are illustrated in Figs. 3 ▸(*a*) and 3 ▸(*b*). Such nonrandom but partially incorrect solutions are often not corrected by *SHELXE*’s density modification and autotracing, as the start fragments dominate the map to be traced. Therefore, the incorrect helices are found and built again every cycle and the process is stuck, despite showing deceptively promising figures of merit and trace extension. Fig. 3 ▸(*c*) shows the lack of progress in the tracing of PDB entry 2nps. This structure contains 308 amino acids in the asymmetric unit and even though the maximum resolution is 2.5 Å, a completeness of only 78%, presumably owing to its anisotropy, suggests that it might rather be considered as a 2.7 Å resolution structure. After three cycles of iterating density modification and autotracing, the weighted mean phase error remains above 70° for a maximum of 142 residues traced, characterized by a misleading high CC of up to 35%.

Even though the presence of reversed helices in the substructure tends to persist throughout tracing, two ways of correcting it became apparent. Running *ARCIMBOLDO_LITE* on more powerful hardware leads to the generation and extension of a larger number of partial solutions. Even if correct and reversed helices at low resolution are not distinguishable from the *Phaser* figures of merit, increasing the pool of substructures generated and trialled allows the correct one to be recognized at the end of the process. Examples of this approach are PDB entries 3miw and 3onx, which were not solved on an eight-core machine but were solved on a 24-core workstation.

An alternative way of tackling coiled coils at low resolution on limited hardware is to generate the corresponding substructures with reversed helices after the placement of several fragments. After rigid-body refinement and rescoring, discrimination of the correct, more complete partial substructures improves, allowing solutions where some of the first fragments had been reversed to be rescued. If combinatorial perturbation of helix direction produces less than 1000 solutions all of them will be explored, otherwise a sparse selection of them will be tried in order to make the number of solutions tractable.

An example within the pool of structures is provided by PDB entry 3miw at 2.5 Å resolution in space group *P*4_2_ and containing ten chains in the asymmetric unit, totalling 432 residues. After a search configured to find ten helices of 30 residues followed by two cycles of density modification and autotracing, a solution was identified that was characterized by 298 traced residues and a CC of up to 35.4%. Its wMPE was 62.9° and it contained 7.9% incorrect trace. Examination of the original solution revealed that of the ten placed helices, two were reversed.

A fresh run with the version of *ARCIMBOLDO_LITE* that probes the helix direction rendered a substructure with all fragments correctly placed. This solution was reached by reversing three of the ten helices during the course of the run. The final solution is characterized by a wMPE of 59.7° for 301 residues, with errors in the trace decreased to 3.7% and a CC of up to 37.8%. Fig. 3 ▸ displays the electron-density map for the partially incorrect (Fig. 3 ▸ *d*) and the correct (Fig. 3 ▸ *e*) solutions. As can be seen from the CC values quoted above, the discrimination between correct and partially incorrect solutions can be narrow; therefore, the coiled_coil mode triggers systematic probing of both helix directions.

#### 3.4.2. *SHELXE* autotracing with helical restraints   

Whereas coiled coils with resolutions of 2.0 Å or better are generally solved using the standard algorithm in *SHELXE*, as the resolution becomes more limited the coverage of the traced model decreases. Electron density in bent areas degrades, leading to extended rather than helical polypeptide traces. As automatic map interpretation stalls, the discrimination of solutions becomes more uncertain. At resolutions of worse than 2.5 Å this often leads to incorrect traces that are nevertheless characterized by a CC above 30%. Avoiding false positives is the reason why *ARCIMBOLDO* has been blocked if the experimental data do not reach this resolution.

A helically constrained main-chain tracing has been incorporated into *SHELXE* (Usón & Sheldrick, 2018 ▸). This choice is automatically triggered within the coiled_coil mode and leads to all autotracing cycles apart from the last being seeded from longer helices and extension of the main chain with helical restraints for Ramachandran angles or helical sliding. The last cycle reverts to *SHELXE* defaults, allowing the tracing of missing nonhelical areas such as loops. The model characterized by the best CC will be kept.

**Figure 4:**
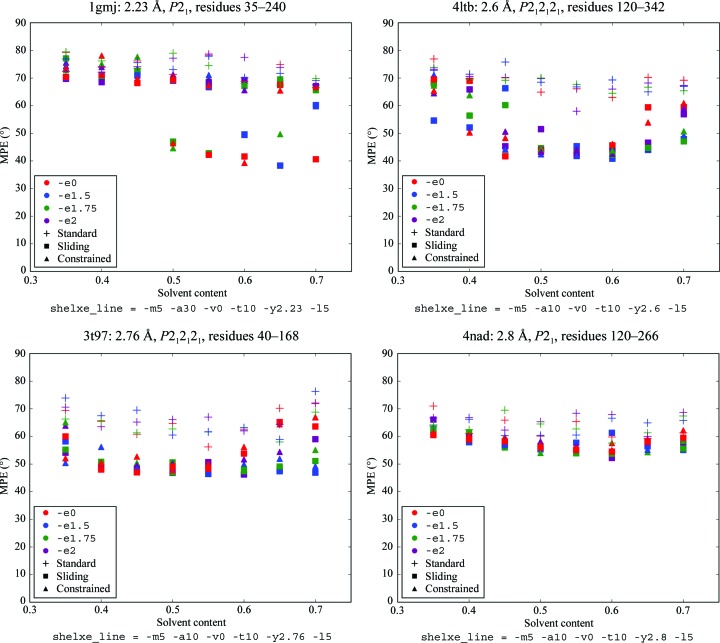

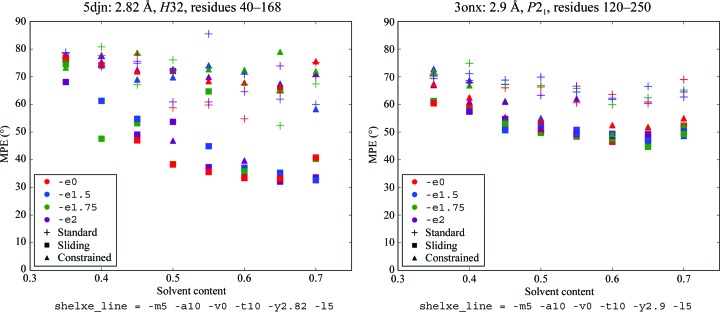
Scatter plots summarizing the results of different parameterizations of three alternative autotracing algorithms in *SHELXE* on six different structures. The colour represents the resolution limit of extrapolated reflections (-e) and the shape represents the autotracing algorithms. In the shelxe_line, -­m sets the number of density-modification cycles, -a the main-chain autotracing cycles, -v the density-sharpening factor, -t the time factor for peptide searches and -y the highest resolution for the starting phases from the model; -I leads to the use of extrapolated reflections in all cycles.

All test structures with resolutions between 2.0 and 3.0 Å were subjected to different parameterizations of *SHELXE* in its standard and constrained autotracing modes in order to derive default parameters for *ARCIMBOLDO* in its coiled_coil mode. Fig. 4 ▸ displays the results of a range of parameterizations on six challenging cases with low resolution and/or a small fraction of the complete structure to start the extension. These graphs show how helically constrained autotracing is decisive in extending the trace and in lowering the weighted mean phase error, allowing a solution to be reached in cases where the standard autotracing would not lead to a solution. While the constrained autotracing (-q8 to -q14) uses larger helical seeds of eight to 14 residues and constraints on the extension of each amino acid to Ramachandran angles in the helical region, the sliding autotracing (-Q) additionally extends the sliding helical fragments of the polypeptide chain and is used by default for coiled coils. Also, *ARCIMBOLDO* usually stops once a solution with CC above 30% has been reached, but in coiled_coil mode it will continue to complete the predetermined number of *SHELXE* expansion cycles.

### 3.5. True solutions, nonrandom solutions and false solutions, and how to distinguish them   

*ARCIMBOLDO*, along with other fragment-based phasing methods, uses the extension of the main-chain trace output by *SHELXE* and the CC characterizing it to identify correct solutions. Cases where the resolution extends to 2 Å or better usually afford a good correlation between the CC of the trace and the wMPE of the structure, and hence a clear-cut discrimination of correct solutions. In such cases, a CC value above 30% typically corresponds to a trace covering over two thirds of the true structure and a map in which side chains can be recognized unequivocally. Exceptions have been observed for false, mistranslated solutions (*i.e.* solutions containing incorrectly positioned helices but in correct orientations). Side-chain assignment in coiled coils tends to be obscured compared with the main chain. Partially correct solutions containing mistranslated or reversed helices may be characterized by high figures of merit more frequently than in other kinds of structures, with the exception of DNA (Urzhumtsev *et al.*, 2016 ▸). Thus, the discrimination of best-scoring incorrect solutions from true solutions was investigated within the pool of test structures.

**Figure 5:**
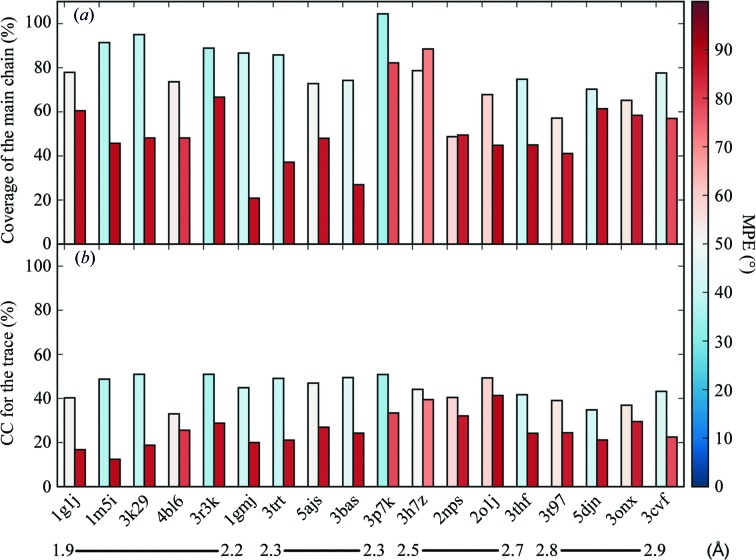
Bar plots representing correct (left) and best-scoring incorrect (right) solutions of the 18 most challenging test cases ordered from high to low resolution. The weighted mean phase error *versus* the deposited structure is colour-coded from red (random) to blue (solved). (*a*) Structure coverage in the trace. (*b*) CC of the trace.

Fig. 5 ▸ shows bars representing the CC and coverage of the traces for correct and best-scoring incorrect solutions for 18 difficult test cases, ordered by resolution. In this graph, correct solutions tend to exceed CC values of 40% and in all cases the correct solution was characterized by a CC at least 4.5% above that of the incorrect solution. At resolutions of 2.5 Å or better both the CC and the percentage of traced residues show a clear-cut difference between correct and incorrect solutions. The situation becomes more complicated as the resolution decreases, especially since the graph compares the correct solution with partially incorrect solutions in which one or more of the helices in the starting substructure were reversed. Such cases include PDB entries 3p7k at 2.3 Å resolution (one reversed fragment), 3h7z at 2.5 Å resolution (two reversed fragments) and 2nps at 2.5 Å resolution (three reversed fragments). Thus, the incorrect solutions compared are not random but are rather mostly correct solutions with some portions traced backwards. Although the trace coverage tends to be significantly higher for the correct solution, this is not true in the case of two of these structures, in which the reversed helix is also extended. Unfortunately, in the absence of the correct solution an increase in CC respective to partially erroneous solutions would not be observed. It is not possible to give an absolute number differentiating both situations, as CC values above 40% have been observed for incorrect solutions, such as PDB entry 2o1j at 2.7 Å resolution. This structure displays true tNCS and could only be solved by accounting for it in *Phaser* as well as placing fragments pairwise. Such pathologies tend to happen in coiled coils and, as seen in Fig. 3 ▸, even in manual building error identification may not be trivial. Therefore, an additional step has been implemented in order to verify the final solution.

### 3.6. Final verification of the best-ranking solution   

Given the concern raised about producing fundamentally wrong solutions bearing good figures of merit, the coiled_coil mode in *ARCIMBOLDO* incorporates an additional step that generates perturbations of the substructure leading to the best solution and compares their scores before and after extension. Combinations of substructures with reversed helices are generated, refined and rescored. There is a hard limit of 1000 combinations, so the sparsity or completeness will depend on the number of fragments. The best-scoring cominbinations in terms of LLG and CC are subjected to extension in *SHELXE*. The idea is that if the discrimination persists or the final solutions are equivalent, confidence in this solution will be justified. Conversely, a warning will be issued if the extension of inconsistent solutions leads to inconclusive results with structurally different structures characterized by comparable figures of merit.

**Figure 6:**
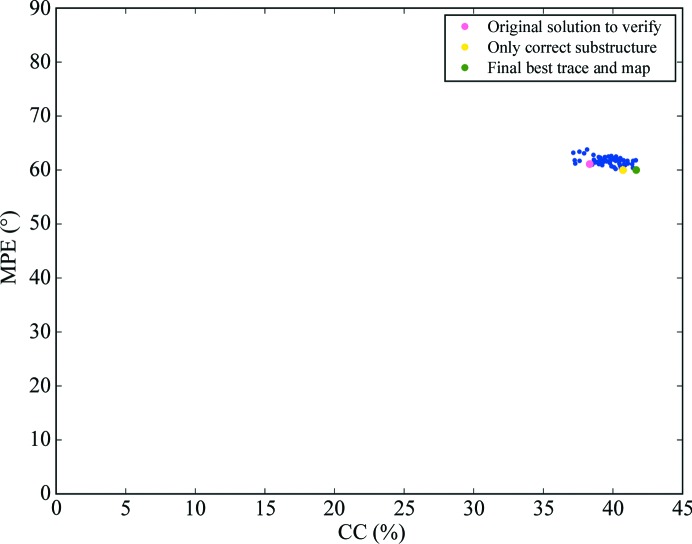
Autotracing results in the case of PDB entry 3miw for the pool of 59 perturbed substructures generated from that giving rise to the solution to be verified, which is also included. All 60 final traces are equivalent and correct. The yellow dot marks the only correct substructure, the green dot the final best trace and map, and the pink dot the new expansion for the substructure generating the originally identified best solution.

This procedure is illustrated by the case of PDB entry 3miw, which is displayed in Figs. 3 ▸(*d*) and 3 ▸(*e*). Taking the *Phaser* substructure that led to the best final CC, a systematic reversal of one, two or three of the ten helices in the substructure was performed, generating a total of 999 additional substructures. Rigid-body refinement and rescoring in *Phaser* was performed and the 60 highest scoring solutions showing LLG values in the range 795.8–678.0 and a *SHELXE* initial CC (INITCC) of 35.11–8.1% were subjected to eight cycles of main-chain autotracing interspersed with density modification. The best-scoring substructure in both LLG and INITCC had all helices correctly placed and oriented, whereas remarkably the substructure leading to the solution to be verified contained two reversed helices. Nevertheless, substructure expansion led to equivalent, correct solutions in all 60 cases, where tracing had reversed the incorrect portions. Thus, the minor differences in CC or wMPE displayed in Fig. 6 ▸ are irrelevant and are derived from slight differences in the extension of the trace and its deviation from the ideal geometry.

## 4. Concluding remarks   

*ARCIMBOLDO_LITE* succeeds in solving 140 out of a pool of 150 test coiled-coil structures with sizes ranging from 15 to 635 residues and resolutions between 0.9 and 3.0 Å on a single workstation. The fragments placed are 1–12 straight polyalanine helices made up of 6–50 amino acids. Run times for *ARCIMBOLDO_LITE* jobs typically take a couple of hours to one day on a single machine with eight physical cores. The successfully solved cases cover the full range of resolution data in the set, from a highest resolution structure at 0.9 Å (PDB entry 1byz) to a lowest resolution structure at 3.0 Å (PDB entry 4qkv). In terms of length and complexity a wide range is covered as well, from a smallest structure with just a single chain in the asymmetric unit comprising 15 residues (PDB entry 1kyc) to a largest structure with four chains in the asymmetric unit totalling 618 residues (PDB entry 2efr).

*ARCIMBOLDO* incorporates a coiled_coil mode, which can be activated by setting this keyword to true in the input file with the extension .bor containing the instructions or selecting it through the *CCP*4 interface. This mode will trigger the following defaults. The otherwise required resolution limit of 2.5 Å in the input diffraction data will be relaxed to 3.0 Å. As r.m.s.d. refinement was required for solution identification in at least in one case, and it was not observed to have negative effects in any case, this calculation will be performed by default. Translational NCS will not be used in fragment placement even if a strong Patterson peak was found, but should be tried by the user if no solution is otherwise achieved. Use of *Phaser*’s packing check during the translation search will output a top solution with acceptable packing. The peak height to accept further translation solutions will be relative to this first well packed solution. The use of helically constrained autotracing in *SHELXE* is required at resolutions worse than 2 Å and is advised in any case for this kind of structure. Leaving the *SHELXE* line unset in the input .bor file will activate *SHELXE* defaults in the coiled_coil mode that differ from the standard defaults as well as from the *SHELXE* defaults. In particular, autotracing will be seeded with longer helices and chains extended only helically during the first iterations. Also, at resolutions worse than 2 Å, after each helix placement generated by *Phaser* complete or sparse combinations of helices reversed in the same positions will be generated, refined and rescored.

Finally, in order to verify the most promising solution, its original substructure will be perturbed by helix reversal and the results of the various extensions compared for evidence of discrimination between groups of consistent solutions.

## Supplementary Material

Supplementary Table.. DOI: 10.1107/S2059798317017582/cb5097sup2.xlsx

## Figures and Tables

**Table 1:** Characteristics of the test sets used in this study

	Test set 1	Test set 2
Range of resolution limits (Å)	0.9–2.9	2.0–3.0
Residues in the asymmetric unit	15–618	45–635
Polypeptide chains in the asymmetric unit	1–4	1–12
No. of different space groups	32	26
Most frequent space groups (and presence in the test set)	*C*2 (13.8%), *P*2_1_2_1_2_1_ (12.8%), *P*2_1_ (10.6%)	*P*2_1_ (17.9%), *C*2 (14.3%), *P*2_1_2_1_2_1_ (7.1%)
Used previously in	Thomas *et al.* (2015 ▸)	

## Appendix A Test structures used in this work

### A1. Test set 1

PDB entries 1byz, 1d7m, 1deb, 1env, 1ezj, 1g1j, 1gmj, 1jcd, 1k33, 1kql, 1kyc, 1m3w, 1m5i, 1mi7, 1n7s, 1nkd, 1p9i, 1s35, 1s9z, 1t6f, 1uii, 1uix, 1usd, 1wt6, 1x8y, 1y66, 1ybk, 1yod, 1zv7, 1zvb, 2akf, 2b22, 2bez, 2efr, 2fxm, 2ic6, 2ic9, 2no2, 2ovc, 2pnv, 2q5u, 2q6q, 2qih, 2v71, 2w6a, 2w6b, 2wpq, 2xu6, 2xus, 2xv5, 2ykt, 2zzo, 3a2a, 3ajw, 3azd, 3bas, 3cve, 3cvf, 3etw, 3h00, 3h7z, 3hfe, 3hrn, 3k29, 3k9a, 3ljm, 3m91, 3mqc, 3ni0, 3okq, 3p7k, 3pp5, 3q8t, 3qh9, 3ra3, 3s0r, 3s4r, 3s9g, 3swf, 3swk, 3swy, 3t97, 3trt, 3twe, 3tyy, 3u1a, 3u1c, 3v86, 3vgy, 3vir, 3vp9, 4dzk, 4dzn and 4e61.

### A2. Test set 2

PDB entries 1kdd, 1pl5, 1t3j, 1u4q, 1unx, 1urq, 1w5h, 2ahp, 2b9c, 2jee, 2nps, 2o1j, 2oqq, 2wz7, 3a7o, 3cyo, 3efg, 3g9r, 3iv1, 3m9h, 3miw, 3nwh, 3onx, 3r3k, 3r47, 3r4h, 3thf, 3tul, 3v2r, 4bl6, 4bry, 4cgc, 4gif, 4hu6, 4l2w, 4ltb, 4m3l, 4n6j, 4nad, 4oh8, 4pn8, 4pn9, 4pna, 4pxj, 4pxu, 4qkv, 4w7y, 4xa3, 4yv3, 5ajs, 5c9n, 5cx2, 5d3a, 5djn, 5eoj and 5jxc.
